# Immune targets for schistosomiasis control identified by a genome-wide association study of African snail vectors

**DOI:** 10.21203/rs.3.rs-5656395/v1

**Published:** 2025-01-03

**Authors:** Michelle Steinauer, Tom Pennance, Jacob Tennessen, Johannie Spaan, Tammie McQuistan, George Ogara, Fredrick Rawago, Kennedy Andiego, Boaz Mulonga, Meredith Odhiambo, Martin Mutuku, Gerald Mkoji, Eric Loker, Maurice Odiere

**Affiliations:** Western University of Health Sciences; Western University of Health Sciences; Harvard University; Western University of Health Sciences; Western University of Health Sciences; Kenya Medical Research Institute; Kenya Medical Research Institute; Kenya Medical Research Institute; Kenya Medical Research Institute; Kenya Medical Research Institute; Kenya Medical Research Institute; Kenya Medical Research Institute (KEMRI); University of New Mexico; Kenya Medical Research Institute

## Abstract

Schistosomiasis, a neglected tropical disease, is transmitted by freshwater snails. Interruption of transmission will require novel vector-focused interventions. We performed a genome-wide association study of African snails, *Biomphalaria sudanica*, exposed to *Schistosoma mansoni* in an endemic area of high transmission in Kenya. Two snail genomic regions, SudRes1 and SudRes2, were significantly associated with snail immunity to schistosomes. SudRes1 includes receptor-like protein tyrosine phosphatases while SudRes2 includes a class of leucine-rich repeat-containing G-protein coupled receptors, both comprising diverse extracellular binding domains suggestive of host-pathogen interaction. Resistant and susceptible haplotypes show numerous coding differences including presence/absence of entire genes. No loci previously tied to schistosome resistance in neotropical snail species showed any association with compatibility suggesting that loci involved in the resistance of African vectors are distinct. Snail ancestry was also strongly correlated with parasite compatibility. These results will inform future efforts to predict and manipulate immunity of a major schistosome vector.

## Introduction

Schistosomiasis is a global scourge, taking a large toll on people who have the fewest resources. Affecting over 260 million people, it is the parasitic disease with the greatest impact on health worldwide after malaria (1, 2). Within the last decade, schistosomiasis control program goals have shifted from reduction of morbidity to elimination or interruption of schistosomiasis as a public health problem by 2030 (1, 3, 4). However, the toolbox with which to combat schistosome transmission has remained virtually the same, dominated by one main approach: mass drug administration (MDA) of praziquantel (5). It is increasingly recognized that in addition to chemotherapy, successful control and elimination will require targeting the aquatic snails which serve as intermediate hosts of the schistosome parasites and transmit them to humans (6, 7). Part of the reason MDA alone is insufficient is that even with effective drug treatment, people become rapidly reinfected by infected snails in the environment (8–10). Schistosomes form chronic infections in snails and continually release hundreds to thousands of infectious stages (cercariae) into the environment daily (11).

Historically, schistosomiasis control programs that are focused on snail control have been the most successful at reducing or eliminating schistosomiasis (12, 13); however, snail-directed control methods are limited and have negative impacts. Molluscicides are indiscriminately toxic and are impractical to apply to vast habitats (12, 14). Furthermore, snail population rebound post-molluscicide application is predicted to increase schistosome transmission (15).

Given the absence of suitable snail vector control methods for contemporary public health interventions, there is a need for new approaches to be developed (16). Genomic and transcriptomic data and resources enable approaches like genome-wide association studies (GWAS) that identify genomic regions of snail vectors involved in resisting schistosome infection (17). Once identified, snail genes and genomic variants associated with resistance to schistosomes could be monitored in wild populations and potentially be manipulated so that snail resistance to schistosomes is enhanced and transmission to humans is interrupted.

The feasibility of manipulating snail resistance to schistosomes may follow similar approaches to engineering resistant hosts for disease control using CRISPR-Cas and associated gene drive technologies (18, 19). However, much of the elegant work regarding transcriptomics and genomics of schistosome-snail compatibility has only addressed these questions in laboratory models of the South American vector of *Schistosoma mansoni*, *Biomphalaria glabrata* (17, 20–24). Very little is known regarding how this body of knowledge will translate to African vectors of *S. mansoni*, through which 90% of *S. mansoni* transmission occurs (25). The recent publication of two African *Biomphalaria* species genomes and transcriptomes (26, 27) provide a path toward molecular-informed snail control in hotspots of transmission.

With the goal of identifying and describing the genetic architecture underlying resistance of African snails to *S. mansoni*, we performed a pooled genome-wide association study (pooled-GWAS) (28) using a wild population of *Biomphalaria sudanica* originating from the shores of Lake Victoria closest to a persistent hotspot of schistosomiasis in Kanyibok, western Kenya (10). We identify two large-effect loci and a strong influence of ancestry on snail resistance to schistosome infection, defined here as complete parasite clearance (including parasite DNA) from snail tissue following exposure. These results reveal how immunogenetics and population demographics contribute to vectorial competence in a natural vector population with direct impact on human health.

## Results

### Pooled-GWAS reveals multiple variants strongly enriched in resistant snails.

Of 1400 F1 *B. sudanica*, whose parents originated from Anyanga Beach (Lake Victoria, western Kenya), exposed to eight freshly hatched *S. mansoni* miracidia from local schoolchildren, 1,109 snails remained in the GWAS study after excluding 254 that died prior to screening for infection and 37 that yielded insufficient genomic DNA (gDNA) quality. The final sample set comprised 615 and 393 snails that were positive (i.e. releasing *S. mansoni* cercariae) or negative (i.e. not releasing *S. mansoni* cercariae nor PCR positive (29)), respectively. Snails that were negative for cercariae but positive for *S. mansoni* gDNA were not considered further. Two equal mass gDNA pools for the pooled-GWAS comprised 493 positive and 295 negative snails. The pooled-GWAS sequencing (Illumina paired-end 150 bp, NovaSeq 6000 S4 flow cell) yielded on average 1.5x coverage per snail (Dataset S1). A total of 4,498,972 variants were retained for analysis. Correlation between sequencing technical replicates of positive and negative pooled gDNA was weak but significantly positive, with 8-fold enrichment for outliers in the top 1% of both replicates (Fig. S1).

**Figure 1:**
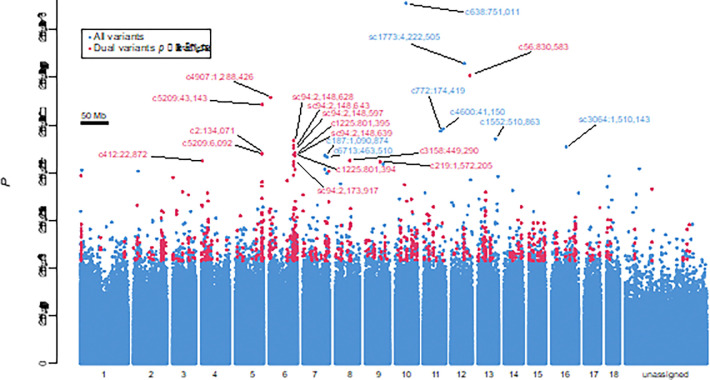
Results of the pooled genome-wide association study (pooled-GWAS) identifying variants associated with resistance to *Schistosoma mansoni* in the *Biomphalaria sudanica* genome. Fisher’s exact test *p* values for all pooled-GWAS variants, are arranged horizontally based on contig orthology to 18 chromosomes (x-axis labels) of the *B. glabrata* genome (xgBioGlab47.1, NCBI RefSeq: GCF_947242115.1) and linkage map analysis (Dataset S2, Fig. S2-S4). Pooled-GWAS dual-variants, defined as two or more proximate (<50 kb and >1.5 kb apart) variants strongly associated with *S. mansoni* resistance (*p* ≤ 2.5e-9) are red. All others are blue. All dual-variants and singleton-variants with p ≤ 1e-17 are labeled red and blue, respectively, with their contig and contig position. Unassigned contigs could not be unambiguously mapped and are mostly small and/or repetitive.

In the pooled-GWAS results, genotype-phenotype association *p* values ranged as low as 1e-30, including 45 variants (0.001%) with *p* ≤ 1e-15 and 1,930 variants (0.04%) with *p* ≤ 2.5e-9 (Fig. 1). Rather than simply defining a genome-wide significance threshold to identify candidates to be validated, we prioritized genomic regions meeting a dual-variant criterion, whereby two or more proximate (< 50 kb and > 1.5 kb apart to ensure support from distinct read pairs) variants are strongly associated with *S. mansoni* resistance (arbitrary threshold of *p* ≤ 2.5e-9); such sliding dual-variant 50 kb windows encompass 18.625 Mb (2%) of the *B. sudanica* reference genome Bs111 (27) and contain 888 (46%) of variants with *p* ≤ 2.5e-9.

### Amplicon panel genotyping reveals population structure and validates variants associated with resistance.

A multiplex amplicon panel was designed using the Genotyping-in-Thousands by sequencing method (30) to genotype variants in individual snails at 234 dual-variants and 12 singleton-variants with *p* < 1e-13 identified from the pooled-GWAS analysis (Dataset S3). The amplicon panel also contained 22 markers for *a priori* gene candidates and 201 ‘neutral’ markers to facilitate a linkage map for improved *B. sudanica* genome assembly (Dataset S2).

An independent set of 122 positive and 98 negative snails not included in the pooled-GWAS were reserved for validation of genomic variants via genotyping with the amplicon panel and are henceforth referred to as genotyped-validation snails. These independent genotyped-validation snails were used to validate the differential allele frequencies associated with *S. mansoni* resistance observed in the pooled-GWAS sequencing data. These were combined with a subset of the pooled-GWAS snails (genotyped-pooled-GWAS snails: 138 positive, 138 negative) for more precise estimates of ancestry and genotype frequencies.

**Figure 2:**
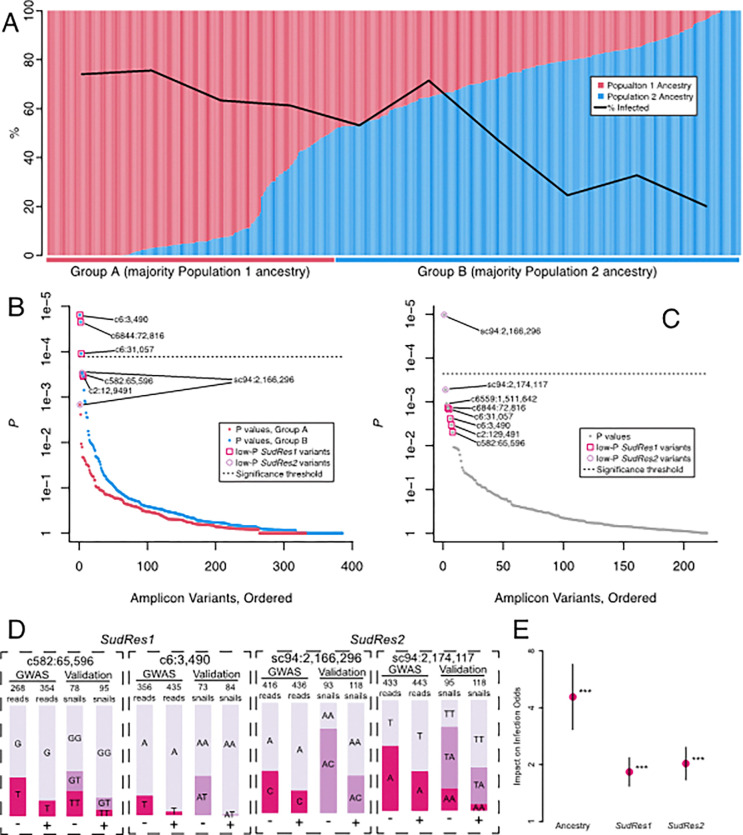
Amplicon panel validation of pooled-GWAS results. (A) Proportion of ancestry from *Biomphalaria sudanica* Population 1 (red) or Population 2 (blue) as predicted by ADMIXTURE (31) is correlated with *Schistosoma mansoni* infection (*p* < 1e-14), such that majority-Population 1 snails (Group A) are 68% positive while the majority-Population 2 snails (Group B) are 41% positive. Total number of samples included in analysis was 503 including 496 genotyped-validation and genotyped-pooled-GWAS *B. sudanica* from this study, 5 inbred *B. sudanica* previously sequenced (27) and 2 outbred *B. choanomphala*. (B) Ordered Fisher’s exact test *p* values per variant of genotyped-validation samples within ancestry groups. *SudRes1*variants c6:3,490, c6:31,057, and c6844:72,816 are significant validated-variants (*p* < 1.3e-04, Bonferroni adjusted significance threshold shown by dotted line) within ancestry Group B (blue dots) and most other top-outliers (1.3e-04< *p* < 1.0e-03) are in *SudRes1* or *SudRes2*. (C) Ordered additive regression *p* values per variant of genotyped-validation samples, after accounting for ancestry. *SudRes2*variant sc94:2,166,296 is a significant validated-variant (*p* < 2.3e-04, Bonferroni adjusted significance threshold shown by dotted line) and most other top-outliers (1.3e-04< *p* < 1.0e-03) are in *SudRes1*or *SudRes2*. (D) Allele and genotype counts for the most significant validated-variants from *SudRes1* (c6:3,490) and *SudRes2* (sc94:2,166,296), which were both dominant markers, and the representative codominant marker variants from *SudRes1* (c582:65,596) and *SudRes2* (sc94:2,174,117), for *S. mansoni* infection positive (+) and negative (−) snails. Values are read counts for each allele in the pooled-GWAS (“GWAS”), and genotype counts in genotyped-validation snails (“Validation”). (E) Means and standard errors for each variable in the best-fitting multiple regression model. Ancestry is the proportion of Population 2 ancestry, and loci *SudRes1* and *SudRes2*are additive effects per each copy of the minor allele at the representative codominant markers c582:65,596 and sc94:2,174,117. ***p < 0.001.

The amplicon panel data (of which median missing data was 2% per locus and 2% per individual) revealed a signal of population structure, evident in both PCA (Fig. S5) and ADMIXTURE (31) analysis (Fig. 2A). With K = 2 ancestral populations (CV = 0.53, versus 0.61 for K = 1), ancestry from Population 2 ranged continuously from 0 to 1 and was significantly correlated with resistance (*p* < 1e-14). Mean Population 2 ancestry was 39% for positives and 62% for negatives. Snails were designated into two groups based on the percentage of their ancestry: 41% of snails had mostly Population 1 ancestry and were deemed “Group A”, while the remainder, with mostly Population 2 ancestry, were deemed “Group B”. Of the snails in Group A, 68% were positive. In Group B, 41% were positive. Thus, any marker differing in frequency between these ancestral populations could be correlated with infection phenotype, even if not linked to an etiological variant, and therefore could explain some outliers identified by the pooled-GWAS. Using markers diagnostic of both groups A and B, we found that *B. choanomphala*, a closely related deep-water taxon/eco-phenotype of *B. sudanica* also collected in Lake Victoria (32), and *B. sudanica* inbred lines originating from Lake Victoria (27), share ancestry with Group A, and thus the GWAS ancestry signal is not caused by interspecies hybridization (Fig. S6). The inferred percentage of Population 1 or 2 ancestry for each snail was similar when estimated using linkage map markers alone, and thus not driven by GWAS outliers (Fig. S7).

After accounting for ancestry, only variants within two genomic regions, henceforth referred to as *SudRes1* and *SudRes2*, showed significance (Bonferroni-corrected *p* < 0.05, designated henceforth as ‘validated-variants’) in a dominance (Fig. 2B) or an additive regression (Fig. 2C) model (Dataset S3). Notably, the p-values of the variants in *SudRes1* and *SudRes2* were amongst the lowest of the dual-variant outliers identified by the pooled-GWAS (Fig. 1). The three validated-variants for *SudRes1* (Fig. 2B) and validated-variant for *SudRes2* (Fig. 2C) acted as dominant markers (only two genotypes observed), so to assess genotype-phenotype associations in more depth we examined codominant proxy variants and used these as representative variants for each region (Fig. 2D). For the *SudRes1* representative variant (c582:65,596, Fig. 2D), allele T was protective in the pooled-GWAS and in the genotyped-validation snails. Combining data from the genotyped-validation and genotyped-pooled-GWAS snails, odds of *B. sudanica* infection with *S. mansoni* were 0.31 for genotype TT, 0.57 for genotype GT, and 1.37 for genotype GG, consistent with an additive effect. For the *SudRes2* representative variant (sc94:2,174,117, Fig. 2D), allele A was protective in the pooled-GWAS and in the genotyped-validation snails. Combining data from the genotyped-validation and genotyped-pooled-GWAS snails, odds of *S. mansoni* infection in *B. sudanica* were 0.36 for genotype AA, 0.74 for genotype TA, and 2.40 for genotype TT, also consistent with an additive effect.

The best-fitting multiple regression model (AIC = 451.11 and *p* < 0.001 for all variables) included: ancestry; *SudRes1* (representative variant c582:65,596, Fig. 2D); and *SudRes2* (representative variant sc94:2,174,117, Fig. 2D), with both genetic markers acting additively (Fig. 2E). The model predicts a ~ 2-fold effect per allele at each genetic marker, and a ~ 4-fold effect of ancestry. Thus, the predicted odds of infection for a snail with no Population 2 ancestry and major allele homozygous genotypes at *SudRes1* and *SudRes2* (4.46) is 62-fold higher (approximately 2^2^*2^2^*4) than the odds for a snail with 100% Population 2 ancestry and minor allele homozygous genotypes at both loci (0.07).

### SudRes1 is rich in paralogous genes encoding MEGF domains.

**Figure 3:**
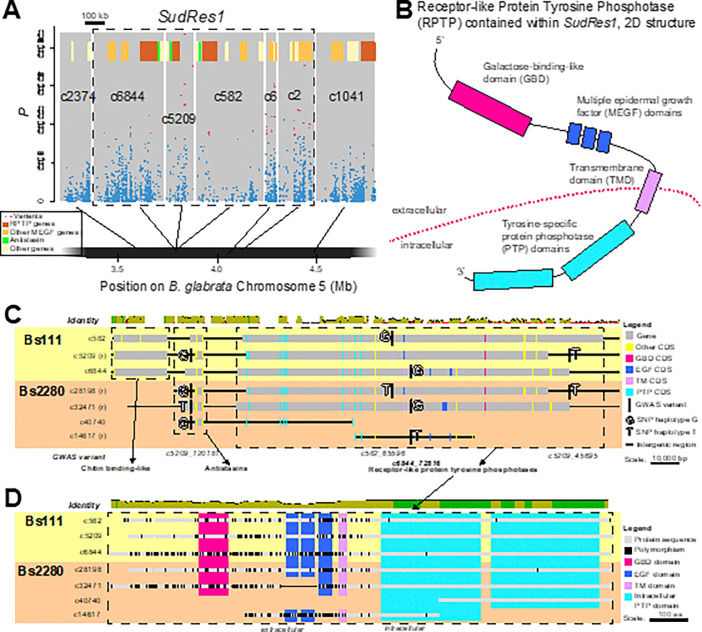
Characterization of *Biomphalaria sudanica SudRes1* genomic region. (A) Pooled-GWAS *p* values in *SudRes1* regions (dashed boxes), which contain pooled-GWAS dual-variants (red) and other variants (blue), defined as in Figure 1. Contigs (grey rectangles) on *B. sudanica* chromosome 5 arranged horizontally based on contig orthology to 18 chromosomes of the *B. glabrata* genome (xgBioGlab47.1, NCBI RefSeq: GCF_947242115.1) and linkage map analysis (Dataset S2, Fig. S2-S4). Gene positions are shown (yellow/brown/green/orange boxes), highlighting particularly prevalent classes of genes: in *SudRes1* the multiple epidermal growth factor (MEGF) and galactose-binding like domain (GBD) containing receptor-like tyrosine-specific protein phosphatase (RPTP), other protein coding genes containing MEGF domains. (B) The predicted protein structure of receptor-like tyrosine-specific protein phosphatase (RPTP) coding gene BSUD.17727 (c6844) present in the *SudRes1* region of the *B. sudanica* genome and containing intronic validated-variants (Fig. S8A). A similar RPTP coding gene is contained within adjacent contigs c582 and c5209 within *SudRes1*, and contig c1041 neighboring *SudRes1* region (Fig. 3A). (C) Nucleotide alignment of paralogous contigs representing a portion of the *SudRes1* region in both Bs111 and Bs2280 genomes, showing location of genes and coding regions relative to GWAS variants used in the amplicon panel. Four G/T polymorphisms are shown, two of which act as non-Mendelian dominant markers due to paralogous amplification, one of which shows two alleles with Mendelian segregation (c582:65,596), and one of which segregates in our population but is invariant in these sequenced genomes (c5209:45,695). Exons are colored by protein domain, and mean pairwise sequence identity is shown at the top (in 100 sliding windows across aligned contigs; green = 100% identity, brown = 30% to <100%, red = < 30%). (D) Amino acid alignment of RPTP genes in *SudRes1*, demonstrating extracellular diversity and loss of functional EGF domains in Bs2280 contig c32471. Protein domains are indicated by color, and non-synonymous polymorphisms in comparison to the majority consensus of paralogs/orthologs are shown in black. Sequence identity is shown as in C, for 25 aa sligning windows in aligned proteins.

*SudRes1* comprises 1.07 Mb of Bs111 and contains 23 protein coding genes across five contigs (c6844, c582, c5209, c6, c2) that are closely linked on chromosome 5 (Fig. 3A, Fig. S2 and Fig. S3). Notably, 10 of these 23 genes encode multiple epidermal growth factor (MEGF) domains (Fig. 3A, Dataset S4). Three of these MEGF proteins in c6844, c5209, and c582, display a common single pass transmembrane domain (TMD) structure, with intracellular tyrosine-specific protein phosphatase (PTP) domains and extracellular MEGF and a galactose binding domain (GBD), forming a receptor-like PTP (RPTP) protein (Fig. 3B). Each of these three RPTP genes within the *SudRes1* region are adjacent to Antistasin-like protein coding genes (Fig. 3C). Only 14 other MEGF/GBD-containing RPTP genes are annotated in Bs111, 13 of which are clustered near *SudRes1* on chromosome 5 (Fig. 3A, Dataset S4).

Of the three validated-variants in *SudRes1* (Fig. 2B), one was contained within the intron of an MEGF/GBD-containing RPTP protein in contig c6844 (Fig. 3C, Fig. S8A), whilst the other two were in the intergenic region either side of another MEGF/GBD-containing protein (contig c6 ortholog 1, Dataset S4). Similarly, the two ‘top-outlier’ variants (defined as variants with 1.3e-04 < *p* < 1.0e-03 following validation, see Fig. 2B) in adjacent contigs c582 and c2 in *SudRes1* were contained within the intronic gene sequence of an MEGF/GBD-containing RPTP protein (c582_65696, Fig. 3C; BSUD.15164, Dataset S4) and another MEGF/GBD-containing protein (c2 ortholog 2, Dataset S4).

Three of the five contigs in the *SudRes1* region, c6844, c5209 and c582, are homologous with each other and match the same unduplicated orthologous region on *B. glabrata* chromosome 5 and *B. pfeifferi* LG5 (Fig. S9 and S10). Aligned read coverage was also atypically low across all *SudRes1* contigs compared to the rest of the genome (Fig. S11).

We compared the Bs111 reference genome, harboring the susceptible *SudRes1* haplotype, to a PacBio genome assembled from a snail homozygous for a resistant *SudRes1* haplotype (Bs2280, coverage of ~ 13x, N50 of ~ 87 kb). While *SudRes1* is not fully assembled in either genome, we can detect substantial structural rearrangements resulting in different numbers of genes for some clusters of homologous loci (Dataset S4, Fig. 3C). Consistent with extensive sequence duplication, Bs2280 includes multiple copies of some amplicon sites (Dataset S6), explaining why these failed to show Mendelian segregation and instead acted as dominant markers. One particularly variable segment, occurring in several divergent copies in both genomes, contains the adjacent antistasin and RPTP genes (Fig. 3C). Among putative orthologs, there are many nonsynonymous differences including occasional differences in protein length resulting in loss of functional domains, especially in RPTPs (Fig. 3D). Notably, this includes EGF domain loss in the resistant snail Bs2280 (Fig. 3D), suggesting that the snails mechanism of *S. mansoni* resistance could involve the loss of function of a protein critical for parasite invasion.

### SudRes2 is characterized by a large family of GRL101-like GPCR genes.

**Figure 4:**
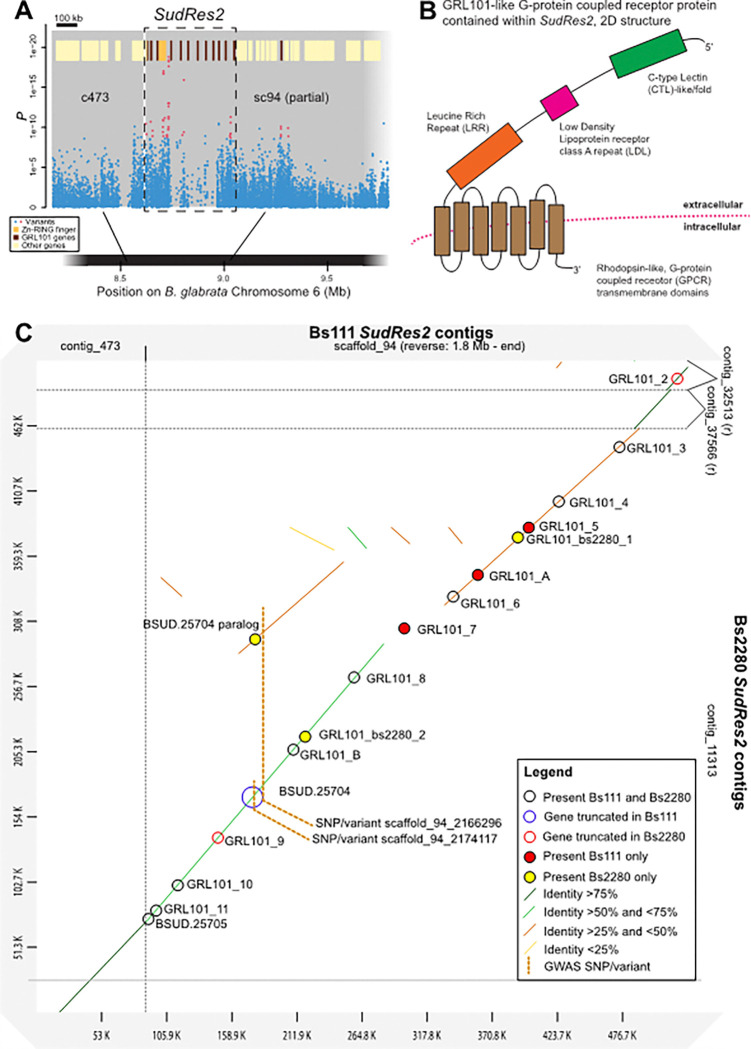
Characterization of *Biomphalaria sudanica SudRes2* genomic region. (A) Pooled-GWAS *p* values in *SudRes2* regions (dashed boxes), which contain pooled-GWAS dual-variants (red) and other variants (blue), defined as in Figure 1. Contigs (grey rectangles) on *B. sudanica* chromosome 6 are arranged horizontally based on contig orthology to 18 chromosomes of the *B. glabrata* genome (xgBioGlab47.1, NCBI RefSeq: GCF_947242115.1) and linkage map analysis (Dataset S2, Fig. S2-S4). Gene positions are shown (yellow/red/orange boxes), highlighting particularly prevalent classes of genes in *SudRes2* encoding a class of leucine-rich repeat containing G-protein couple receptors (GRL101) with C-type lectin and low-density lipoprotein extracellular domains (partial GRL101 genes included), and a Zinc-RING finger and inhibitor of apoptosis containing protein. (B) A representative predicted protein structure of a GRL101-like G-protein coupled receptor coding gene, twelve of which were predicted through manual annotation within the *SudRes2* region of contig sc94 (1.82–2.26 Mb) in the *B. sudanica* genome. (C) Dot plot constructed using D-GENIES (69) comparing synteny of the *SudRes2* region in *Biomphalaria sudanica* Bs111 genome (27) and the Bs2280 resistant snail genome, highlighting regions of divergence and structural rearrangements between the two genomes. GRL101 genes are indicated along with Zn-RING-IAP gene *BSUD.25704*and its paralog.

*SudRes2* comprises a 440 kb region between 1.82 and 2.26 Mb on contig sc94 of Bs111 chromosome 6 (Fig. 4A, Fig. S2 and Fig. S4). Following manual annotation of *SudRes2*, 14 protein coding genes were identified (Fig. S12 and Dataset S5). Ten of these encode mutually paralogous GRL101-like proteins, defined as G-protein coupled receptor (GPCR) transmembrane proteins with extracellular regions containing a leucine rich repeat (LRR) region, a low-density lipoprotein receptor class A repeat (LDL) and a C-type lectin-like (CTL) domain (Fig. 4B); an additional two GRL101-like genes in *SudRes2* are missing the CTL or LDL domain and may represent incomplete proteins (Dataset S5). Of the 437 GPCR genes in Bs111 (27), only six genes outside of *SudRes2* are annotated as possessing GRL101, CTL, and LDL domains (Dataset S5).

Bs2280, the resistant snail genome, was also homozygous for a resistant SudRes2 haplotype, facilitating comparison with the susceptible Bs111 haplotype. In both genomes, assembly of this region is nearly complete, and reveals several genes that are present in only one genome, or are duplicated in one haplotype (Fig. 4C). Similarly, some orthologs differed in length between genomes, being truncated in one or the other.

Both the validated-variant and top-outlier variant in Bs111 *SudRes2* are contained in the non-coding regions of non-GRL101 gene, *BSUD.25704*, clustered within the GRL101 genes, which when complete encodes a protein with a zinc finger RING-type (Zn-RING) domain and inhibitor of apoptosis (IAP) repeat region (i.e. Zn-RING-IAP) (Fig. 4C, Fig. S8B). In the reference/susceptible *SudRes2* haplotype, a nonsense variant (Bs111 sc94:2,167,458) in *BSUD.25704* truncates the protein at 323 aa, however in the resistant Bs2280 ortholog, a 391 aa protein can be translated. Furthermore, in Bs2280 a paralogous Zn-RING coding gene (truncated and not including IAP) is present within a divergent portion of the orthologous *SudRes2* region (Fig. 4C). Amplification of both *BSUD.25704* and its paralog in resistant haplotypes is likely responsible for the non-Mendelian behavior of the validated marker (sc94:2,166,296) which appears as heterozygous in resistant snails (Fig. 4C; Dataset S6). On the boundary of *SudRes2* is a baculoviral IAP repeat containing (BIRC) protein coding gene *BSUD.25705* (Fig. 4C), many of which are contained in the genome regions neighboring *SudRes2*.

Prior to the manual annotation of the 14 genes contained with the Bs111 genome, the *SudRes2* region was exceptional in that: 1) only four protein coding genes in 440 kb had been annotated in this region of the reference *B. sudanica* genome, much lower than the genome-wide density of one gene per 40 kb; 2) a low density of variants were present (Fig. 3A and Fig. S12), and; 3) a large drop in aligned pooled-GWAS read coverage across the central 240 kb of *SudRes2* was apparent (Fig. S13 and S14). To confirm the presence and validity of the manually annotated GRL101 genes, *B. sudanica* RNA transcript data was successfully aligned to 11 of the 12 predicted GRL101 CDS sequences in the *SudRes2* region (all except GRL101_3). Phylogenetic analysis of protein coding sequences also revealed that the *B. sudanica* syntenic (conserved gene order) orthologs identified in *B. glabrata* and *B. pfeifferi* (Dataset S5) were also the most closely related (Fig. S15).

## Discussion

### Identification of loci associated with schistosome resistance in a wild snail vector population.

In this study, we identified and validated two previously uncharacterized genomic regions, *SudRes1* and *SudRes2*, in the African snail vector *B. sudanica* that are associated with resistance to *S. mansoni* infection, each contributing a similar effect size of a ~ 2-fold change in *S. mansoni* infection odds ratio per allele. Both regions contain long segments with unusually low pooled-GWAS read coverage and contain few annotated genes in the reference genomes of *Biomphalaria* sp., suggesting possible structural variation or allelic divergence that preclude unambiguous alignment of reads and complicates assembly and annotation of these regions. It is crucial therefore to acknowledge that the validated-variants associated with schistosome resistance may not themselves be causal polymorphisms, instead they highlight that something significant is occurring in these regions that may remain elusive using the current *B. sudanica* genome assemblies (27), potentially due to structure rearrangements or unaligned alleles. Manual annotation of both *SudRes* regions revealed that they are enriched with transmembrane protein coding genes with diverse extracellular regions composing of protein-protein interacting and carbohydrate binding domains, relevant to immune-related functions such as pathogen recognition (33, 34).

*SudRes1* is characterized by MEGF-domain containing genes, including receptor-like protein-tyrosine phosphatases (RPTPs), comprising extracellular MEGF, extracellular GBD, and intracellular tandem PTP domains. These potentially heavily glycosylated RPTPs may form stable dimers on the cell surface (35), with ligand binding triggering confirmational changes that expose or occlude catalytically active regions of the intracellular membrane-proximal PTPs, transducing signals across the cell membrane (36, 37). The presence of schistosome resistance-associated variants surrounding the *B. sudanica* RPTPs suggests that increased efficacy or upregulation of these proteins may counteract *S. mansoni*-induced phosphorylation, one of the parasite’s strategies to manipulate or evade the snail immune system and promote its survival (38). Neighboring each RPTP in the *SudRes1* region were antistasin genes, a type of serine protease inhibitors that were originally described as anticoagulants in blood feeding species and since been attributed to immune responses in marine gastropods (39). While *SudRes1* is not fully assembled in either Bs111 and Bs2280 genome, it appears likely that homologous contigs within each genome represent paralogous segments rather than alleles, since similar gene counts were observed in both genomes, suggesting recent gene duplications after *B. sudanica* diverged from *B. glabrata* and *B. pfeifferi*. The *SudRes1* region was previously noted as showing exceptionally high nucleotide diversity in *B. sudanica* (27), which when coupled with the pooled-GWAS results suggest that pathogen-mediated balancing selection may act on these genes as previously hypothesized. Our findings here support the approach of using genome hyperdiversity as a proxy for identifying immune related genes in uncharacterized genomes (27).

*SudRes2* contains resistance-associated variants within a Zn-RING-IAP gene, which neighbors a cluster of 12 leucine rich repeat-containing G protein-coupled receptor (LGR) family genes where pooled-GWAS variants are distributed throughout. The structure of the *SudRes2* LGR proteins is similar to GRL101, a LGR first described in the gastropod species *Lymnaea stagnalis* notable for its N-terminal extracellular LRRs and LDLs (UniProt accession P46023 (40)). Unique to the *B. sudanica* GRL101 genes characterized in *SudRes2*, however, is the N-terminal C-type lectin (CTL) fold/domain. CTL domain containing proteins are established components of both vertebrate and invertebrate innate immune systems as recognition and effector molecules, which show pathogen dependent expression patterns (41–43). Due to the architecture of the *SudRes2* GRL101 proteins, the CTL domain likely extends away from the cell membrane exposing the CTL binding region to cytoplasmic ligands (such as those derived from invading pathogens) that could then be presented to the GPCR membrane-spanning binding pocket, triggering G-protein activation. Homology and phylogenetic placement of the syntenic GRL101 proteins indicates a shared ancestry, and possible functional conservation, in GRL101 genes retained since the split of *B. glabrata* and African *Biomphalaria* species ~ 5 Mya (26, 44), although the incomplete assembly of available *Biomphalaria* genomes in this hyperdiverse region may impede inferences of expansion and contraction. To our knowledge, GRL101-like proteins have not been affiliated with immunity in gastropods, but have been shown to play an important role in innate immunity of other invertebrates (45, 46). Although GRL101-like genes were present elsewhere in the *B. sudanica* genome, the dense cluster of GRL101 genes in the *SudRes2* region is unique in that in Bs111 it is the only region < 0.5 Mb with 12 GRL101 genes, with the caveat that GRL101 genes elsewhere in the *B. sudanica* reference genome may also not be annotated correctly.

### Evidence of a shifting snail population structure in Lake Victoria could lead to increased infections.

A surprising result was the discovery of ancestry heterogeneity in our GWAS snails, whose parents had all been collected at the same time and place. More remarkable still, this ancestry signal is strongly correlated with schistosome resistance. Thus, many outliers in our pooled-GWAS could represent ancestry-informative markers with no physical linkage to resistance genes. Population ancestry estimates using only neutral linkage map markers were very similar to those using the full panel including GWAS outliers, supporting that the ancestry effect observed is real and not an artifact of using atypical variants implicated by the GWAS. Considering the importance of snail ancestry in schistosome compatibility here, potential causes behind the population structure were tested. First, no support for reproductively isolated cryptic *Biomphalaria* species in Lake Victoria causing the structure was found, since the ancestry estimates varied continuously between populations, and because no marker was fixed between ancestral populations. Second, since the estimated allele frequencies for the two ancestral populations are continuously distributed, and only few alleles are observed at a similar frequency, we do not expect that a single prolific snail had parented a disproportionate amount of the offspring used in the GWAS. Third, while *B. sudanica* and the deep-water taxon *B. choanomphala* are closely related, perhaps being ecophenotypes (32), sympatric, and distinct in parasite susceptibility (47), they do not represent the ancestry groups and cluster with *B. sudanica* having a majority Population 1 ancestry. Rather, Population 2 *B. sudanica* is distinguished by a set of alleles that do not appear to be common in either species.

The population structure of *B. sudanica* in Lake Victoria observed in our results is more consistent with historical isolation and reconnection of populations. Of the major lakes in the Albertine Rift Valley lake system (Victoria, Tanganyika, Malawi), Lake Victoria is a relatively young lake forming ~ 0.4 Mya, and has gone through at least three major desiccations in the past 100,000 years (48–50). Shaped by such drought events, the cichlids of Lake Victoria have become a famous study system due to the astounding levels of explosive diversification that has occurred since the last desiccation event < 15,000 years ago (51, 52). The population structure of *B. sudanica* observed suggests that indeed cryptic population structure is present, potentially caused by these historic events, yet the degree of admixing between populations in our study signifies ongoing outcrossing rather than clear speciation. The signatures of ongoing admixing may be influenced by hydrologic patterns of Lake Victoria. The collection site was ~ 20 miles north of the Rusinga channel connecting the open lake and narrow Winam gulf. The Winam gulf is a unique lake environment given that it is somewhat separated from open lake water due to the prevailing currents limiting circulation of water (53, 54), is comparatively shallower, potentially exacerbating historic water level changes, and has more protected shores, providing different freshwater habitats than those present in the open lake. The hydrology of the Rusinga channel and therefore Winam gulf was most recently disrupted by the blocking of the Mbita passage in the early 1980’s, until its unblocking in 2017 (55), therefore occurring just prior to our snail collections in early 2018. The return of north-easternly flow of open-lake water into the Winam gulf through the Mbita passage has caused a shift in both bacterial and planktonic communities in the Winam gulf (56, 57), and may have allowed dispersal of *B. sudanica* populations on floating vegetation, such as water hyacinth between lake areas (58). Although we cannot establish the potentially different geographic origins of the *B. sudanica* representative of each population using currently available data, these snail population differences, and therefore vectoral competency differences, could explain why some locations around lakes are persistent hotspots of transmission while others are not (10). These findings underscore how pathogen resistance can vary substantially between closely related populations, and in this instance could suggest that schistosome transmission may be more persistent in lake regions where highly susceptible *Biomphalaria* populations, i.e. majority Population 1 ancestry, are present.

### Evolutionary dynamics of snail-schistosome interaction.

This study complements extensive work on immune mechanisms in laboratory populations of *B. glabrata* (17, 20, 22, 59), facilitating comparisons between snail species. Notably, there was no overlap between our validated GWAS hits and loci linked to resistance in *B. glabrata*. The amplicon panel included at least two amplicons within or near each of these *a priori* candidates (27), and none of them showed a significant association with infection phenotype. *SudRes1* resides on chromosome 5, the site of a large resistance QTL in *B. glabrata* (22), though about 10 Mb away and thus unlikely to include the same gene(s). *SudRes2* resides on *B. sudanica* chromosome 6, which contains a density of schistosome-resistance *a priori* loci including *PTC1* (59), *tlr* (60), *sod1* (61), and the closest, *prx4* (62), at ~ 1 Mb away is not likely to be responsible for association in our analysis. While allelic variation in orthologs of *B. glabrata* resistance loci are not associated with *S. mansoni* resistance in *B. sudanica*, reverse genetics approaches successfully applied in *B. glabrata* (63, 64) can be used in the future to functionally evaluate the roles of these genes. *Biomphalaria sudanica* may rely on entirely different genetic mechanisms for parasite resistance than *B. glabrata*. However, considering the extensive genotype-by-genotype interaction documented between *B. glabrata* and *S. mansoni* (65), mediated by hyperdiverse resistance loci suggestive of long-term balancing selection (17, 59), we propose a more nuanced scenario. Namely, that resistance alleles fluctuate dynamically in response to the genotypes of local parasites (including other trematodes), so the loci harboring large-effect, intermediate-frequency alleles will vary over time and space, even within a species. Other trematode species may be more prevalent and exert greater selection pressure on these snails, indirectly impacting resistance to *S. mansoni*. The striking 4-fold effect of ancestry on odds of infection also supports this dynamic view, as subpopulations different in resistance may be distributed unevenly across Lake Victoria.

Diversity at both *SudRes1* and *SudRes2* is high, as shown by previous polymorphism scans (27) and confirmed by the substantial sequence and structural divergence between susceptible genome Bs111 and resistant genome Bs2280. We are unable to pinpoint causal genes yet, and the numerous differences between resistant and suspectable haplotypes means several candidates are plausible. Each genome assembly contains genes and/or gene segments that are absent in the other assembly. Thus, the host-parasite interaction mechanism(s) could include recognition by *S. mansoni* of snail hosts possessing a particular susceptibility protein, triggering successful infection, or else recognition of the parasite by snails possessing a particular resistance protein, triggering immune cascades (66). If what matters is parasite recognition of the host, loss of EGF domains in *SudRes1* RPTP proteins in resistant snails may inhibit parasite recognition of snail host molecules, and hinder parasite-driven modification of the host response. Similarly, loss of *SudRes2* GRL101 genes in resistant snails may prevent the parasite from recognizing these snails, rendering them immune. In contrast, if resistance is driven by host recognition of the parasite, this could be mediated by nonsynonymous allelic differences in *SudRes1* and *SudRes2* or larger structural changes. For example, at *SudRes2* the non-truncated Zn-RING-IAP gene *BSUD.25704*, and its additional partial paralog, could fulfill a recognition function in resistant but not susceptible genomes. Parasite-resistance regions *PTC1* (59) and *PTC2* (17) in *B. glabrata* can show both dominant resistance and dominant susceptibility (59, 67) and are also highly polymorphic in *B. sudanica* (27), indicating a similar pattern of immune-relevant balancing selection consistent with long-term snail-parasite coevolution.

By revealing immune-relevant genetic variation in *B. sudanica*, the primary vector in African Great Lakes, this work represents an important step toward molecular-informed vector control to combat schistosomiasis in high-transmission global regions, including gene drive technologies (68). However, we demonstrate that genetic manipulation of snails for schistosomiasis control will require navigating the ever more complex genetic architecture of snail resistance, particularly when considering the non-overlap in findings from the laboratory model South American species *B. glabrata* and the diversity of snail vector species responsible for the majority of schistosome transmission in Sub-Saharan Africa. The impact of any resistance allele may vary due to genetic background and environmental factors. However, we expect that the large-effect loci identified here will play key roles in the continued elucidation of snail immunity as it pertains to human disease.

## Materials and Methods

Additional description of materials and methods is provided in SI Materials and Methods.

## Data Availability

All sequence data, including PacBio HiFi raw reads from genome sequence data of *B. sudanica* Bs2280, has been uploaded onto the NCBI SRA under BioProject PRJNA1149315 with BioSample accessions SAMN43241892, SAMN43241893, SAMN43241894, SAMN43241895, SAMN45084274. The genome assembly of Bs2280 is available on figshare, DOI: 10.6084/m9.figshare.27985880.
